# Chitosan nanofiber biocomposites for potential wound healing applications: Antioxidant activity with synergic antibacterial effect

**DOI:** 10.1002/btm2.10254

**Published:** 2021-09-16

**Authors:** Mitra Bagheri, Majid Validi, Abolfazl Gholipour, Pooyan Makvandi, Esmaeel Sharifi

**Affiliations:** ^1^ Department of Microbiology and Immunology School of Medicine, Shahrekord University of Medical Sciences Shahrekord Iran; ^2^ Department of Medical Laboratory Sciences School of Allied Medical Sciences, Shahrekord University of Medical Sciences Shahrekord Iran; ^3^ Cellular and Molecular Research Center Shahrekord University of Medical Sciences Shahrekord Iran; ^4^ Istituto Italiano di Tecnologia Centre for Micro‐BioRobotics Pisa Italy; ^5^ Department of Tissue Engineering and Biomaterials School of Advanced Medical Sciences and Technologies, Hamadan University of Medical Sciences Hamadan Iran

**Keywords:** antibacterial, antioxidant, chitosan/polyethylene oxide nanofibers, electrospun, silver nanoparticles, wound healing, zinc oxide nanoparticles

## Abstract

Bacterial wound infection is one of the most common nosocomial infections. The unnecessary employment of antibiotics led to raising the growth of antibiotic‐resistant bacteria. Accordingly, alternative armaments capable of accelerating wound healing along with bactericidal effects are urgently needed. Considering this, we fabricated chitosan (CS)/polyethylene oxide (PEO) nanofibers armed with antibacterial silver and zinc oxide nanoparticles. The nanocomposites exhibited a high antioxidant effect and antibacterial activity against *Staphylococcus aureus*, *Escherichia coli*, and *Pseudomonas aeruginosa*. Besides, based on the results of the cell viability assays, the optimum concentration of ZnONPs and AgNPs in the nanofibrous mats is 0.2% w/v and 0.08% w/v respectively and had no cytotoxicity on fibroblast cells. The scaffold also showed good blood compatibility according to the effects of coagulation time. As well as significant fibroblast migration and proliferation on the wound margin, according to wound‐healing assay. All in all, the developed biocompatible, antioxidant, and antibacterial Ag‐ZnO NPs incorporated CS/PEO nanofibrous mats showed their potential as an effective wound dressing.

## 1 INTRODUCTION

Wound infection is a common and unsolved issue in preventing wound healing. Any kind of wound on the skin is susceptible to the accumulation of infectious bacteria.^1^ With the spread of antibiotic‐resistant bacteria, employing antibiotics to prevent and treat wound infections is inefficient. In addition, it imposes extra costs on the patient and society. Consequently, alternative materials are pivotal to combat pathogens.^2^, ^3^

Advanced wound dressing is the barrier to care for sores and should mainly target bacterial infection by sufficient sealing of the injury microenvironment from peripheral contaminants. Simultaneously, they can eradicate excessive exudate, enhance autolysis debridement, and maintain adequate water for healing. In addition to barrier property, they would also have specific flexibility, applicability, constancy, biodegradability and be able to remove, pace the healing process, and decrease the risk of infection.^4^, ^5^ Today, the use of advanced mats comprising antibacterial properties is essential in preventing and treating wound infections. Despite the diversity of wound dressings in the market, highly exuding wounds are difficult to heal as dressings cannot efficiently inhibit the wound's microbial invasion.^6^ Antimicrobial dressings involve incorporating an antiseptic agent comprising zinc oxide, silver, titanium oxide, and iodine, adding to the dressings to avoid microbial contamination.^7^ Electrospinning is one of the most common methods to produce electrospun wound dressing mats.^8^, ^9^ By fabricating nanofibers with the appropriate surface nanotopography, density, and two‐dimensional structure, electrospinning is an effective way to fabricate substrates targeted for tissue engineering.^10^ These nanofibrous mats can be employed as a wound dressing because of their high porosity, making them suitable for ventilation and air exchange. Since the skin is repaired in a moist environment, the porous structure of mats can provide an environment ideal for healing through the exchange of gas.^11^

Chitosan is a cationic biopolymer with a linear structure derived from hydrolysis of the natural polymer chitin.^12^ Due to its biocompatibility, biodegradability, cell transplantation ability, along with antibacterial and antifungal properties, it is widely deployed in scaffolds for regenerative medicine applications and wound healing.^13^, ^14^ Due to its natural origin and wound healing capability, chitosan wound management products, for example, ChitoSorb (ChitoTech), KytoCel (Aspen Medical), ChitoGauze Pro (HemCon), and Opticell (Medline), used to treat severe cases of burned skin, open and deep wounds.^15^ Besides, nanofibrous chitosan scaffolds play a significant role in wound healing due to their positive influence on the re‐epithelialization and regeneration of the granular layer of the wounds.^15^, ^16^ Nonetheless its application for wound dressing has some limitations, for example, poor mechanical strength and low antibacterial activity, which is inadequate for effective wound dressing.^17^

Recently, there has been a growing interest in research on nanofibrous scaffolds developed by electrospinning of natural and synthetic polymers solely or as blend with bioactive plant extracts and metallic nanoparticles for tissue regeneration.^18^, ^19^ Electrospinning of chitosan is very difficult because of the cationic nature of the solution, its rigid chemical structure, and its specific intermolecular interactions.^14^, ^20^The simplest and most effective way to enhance the electrospinning capabilities of chitosan is blending with another polymer (e.g., polyethylene oxide) with high electrospinning potential.^21^, ^22^ Polyethylene oxide (PEO) is a biocompatible, noncytotoxic, and hydrophilic polymer and is widely used in biomedical applications. Moreover, PEO is employed in combination with chitosan to form uniform nanofibers with suitable mechanical properties for wound healing and tissue engineering applications.^23^, ^24^

Silver nanoparticles are the most prevalent nanostructures for antimicrobial therapy due to their potent microbicide properties.^25^ Ag nanoparticles have been employed in numerous approaches for wound treatment, such as impregnation of silver nanoparticles in polymeric bandages for upgrading of the existing polymer‐based moist wound dressings or adding of silver ions in hydrocolloids, foams (PolyMem Silver), hydrogel (SilvaSorb gel), alginate dressings and silver sulfadiazine (SSD) creams and gauze dressings (Urgotul® SSD, Askina Calgitrol Ag [silver alginate], Acticoat™). For instance, Argovit™ is a silver nanoparticle formulation with antimicrobial activity, showing neither cytotoxic nor genotoxic effects in human lymphocytes, and promotes diabetic wound healing.^6^, ^26^ Porous and nanofibrous antibacterial materials of the nanosilver/cellulose composite showed excellent antibacterial properties as a result of the Ag nanoparticles. The composite alsoimproved infected wound healing owing to the absorbing capability for wound exudate besides promoted keratinocyte proliferation and growth, thus providing an appropriate environment for cell growth.^25^, ^27^ Other antimicrobial nanocomposite scaffolds containing Ag NPs were blended with polymers such as α‐chitin/β‐chitin hydrogel, alginate/hydroxyapatite, poly(lactic acid), poly(ɛ‐caprolactone), and poly(3‐droxybutyrate‐*co*‐3‐hydroxyvalerate)^25^ for tissue engineering applications.

Antimicrobial properties of low concentration of ZnO NPs and their role in fibroblast proliferation, angiogenesis, and increased re‐epithelialization properties make them an active ingredient in wound dressings.^7^, ^16^, ^27^, ^28^, ^29^ It was reported that the bactericidal activity of zinc oxide NPs against *Streptococcus mutans* is significantly higher than that of Ag NPs at both 1% w/w.^25^ Blending ZnO NPs with chitosan increases microbicidal capability and increases collagen deposition in the wound area.^16^

Altogether, the purpose of this study was to design antibacterial and hemocompatible chitosan/PEO‐based wound dressings using the electrospinning technique. In this regard, silver and zinc oxide nanoparticles were employed to enhance the antibacterial capability of the nanofibers. We then evaluated the wound healing capability, cytotoxicity, and hemocompatibility of the nanofibrous mats. As the medical applications of metal‐based nanomaterials are associated with their cytotoxicity, many efforts have been committed for diminishing the cytotoxicity of nanometals. Different strategies can be deployed to decrease the cytotoxicity of metallic nanoparticles, such as immobilization of nanoparticles in polymeric matrices/nanocarriers or surface coatings. For instance, chitosan, dextran, chitosan/alginate beads, polyethylene glycol, silica, or titania prepare unique materials with new characteristics to both components, e.g., tensile strength, flexibility, and biodegradability.^7^, ^25^

Combining biopolymers with nanofillers is an alternative for achieving materials with improved antibacterial properties.^27^ Antioxidant are suggested to assist the management of wound oxidative stress and, thus, accelerate wound healing.^30^ Therefore, we proposed electrospun nanofibrous mats of Cs/PEO/ZnONPs and AgNPs that possess antibacterial and antioxidant activity as promising candidates for wound healing.

## 2 RESULTS

### 2.1 MIC determination

The antibacterial effects of silver and zinc oxide nanoparticles on common wound infection bacteria were investigated to achieving a proper and effective concentration of nanoparticles against Gram‐positive and Gram‐negative bacteria, which should be incorporated into nanofibers to achieved nanofibrous mats with suitable antibacterial properties. In this regard, MICs of different concentrations of silver nanoparticles and zinc oxide nanoparticles and control antibiotics are determined against *Staphylococcus aureus*, *Escherichia coli*, and *Pseudomonas aeruginosa* (Table 1).

**TABLE 1:** MIC determination of AgNPs, ZnONPs, and control antibiotics against *Staphylococcus aureus*, *Escherichia coli*, and *Pseudomonas aeruginosa*

Bacteria	Control (μg/ml)	Silver nanoparticle (μg/ml)	Zinc oxide nanoparticle (μg/ml)
*S. aureus*	P: 16	50	61.87
*E. coli*	AM: 4	25	8100
*P. aeruginosa*	GM: 4	50	2500

Abbreviations: AM, ampicillin; GM, gentamicin; P, penicillin.

In the present study, the MIC concentrations for *E. coli* and *S. aureus* were 25 and 50 μg/ml, respectively, consistent with the previous study results. In line with the present study results, the MIC results of the study by Aragão et al. also showed that the antibacterial effects of silver nanoparticles against *E. coli* are more significant than *S. aureus*.^31^

### 2.2 Characterization

**FIGURE 1:**
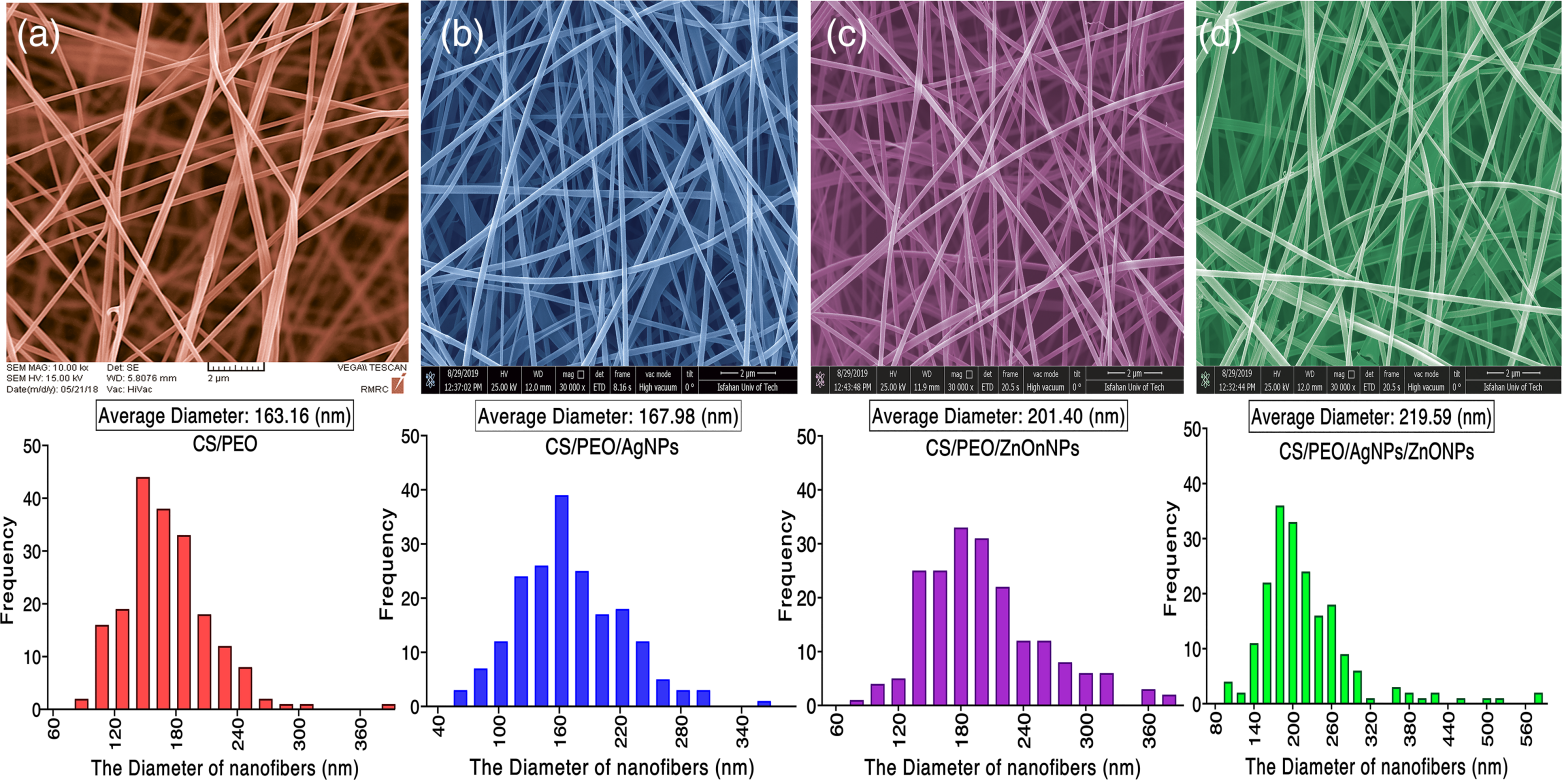
Morphological characterization of the prepared electrospun mats by FESEM and fiber diameter distribution of (a) Bare CS NFs; (b) CS NFs adorned with AgNPs; (c) CS NFs adorned with ZnONPs; (d) CS NFs adorned with AgNPs‐ZnONPs. The scale bar is 500 nm. AgNPs, silver nanoparticles; CS, chitosan; NF, nanofiber; SEM, scanning electron microscopy; ZnONPs, zinc oxide nanoparticles

The surface morphology of the nanofibrous mats assessed using FESEM is shown in Figure 1. Uniform fibers 100–300 nm in diameter were obtained for nanofibrous mats containing silver and zinc oxide nanoparticles suitable for fibroblast cell attachment. The optimal diameter of chitosan/PEO nanofibers has been reported between 120 and 230 nm.^32^ In the present study, based on FESEM results, the average diameter of electrospun nanofibers containing AgNPs, ZnONPs, and AgNPs‐ZnONPs was 185 ± 15 nm, 184 ± 26 nm, and 193 ± 15 nm, respectively.

**FIGURE 2:**
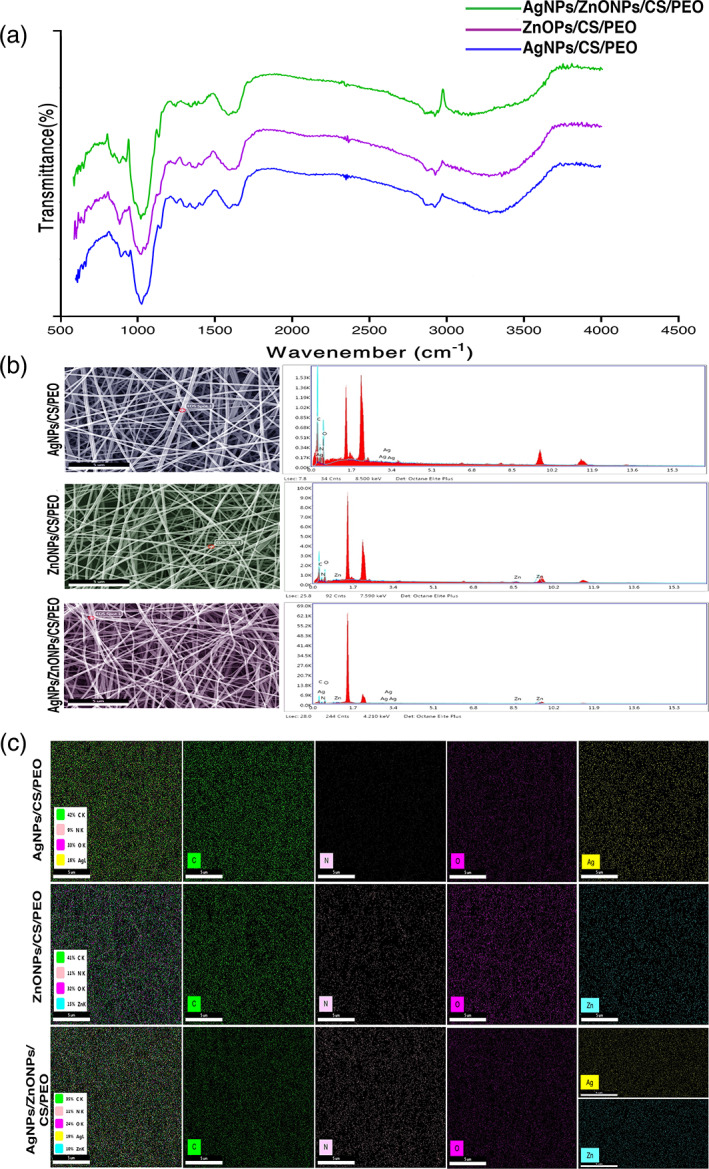
(a) FTIR spectra of CS/PEO/AgNPs, CS/PEO/ZnONPs, and CS/PEO/AgNPs/ZnONPs. (b) Spotted analysis of the AgNPs‐CS, ZnONPs‐CS, AgNPs‐ZnONPs‐CS mats: The results confirm the presence of AgNPs and ZnONPs in mats. AgNPs, silver nanoparticle; CS: chitosan. (c) Map analysis of the AgNPs‐CS, ZnONPs‐CS, AgNPs‐ZnONPs‐CS mats: the presence and distribution of the elements in the mat were confirmed. The percentage of these elements was also determined. Ag, silver; AgNPs, silver nanoparticles; C, carbon; CS, chitosan; N, nitrogen; O, oxygen; Zn, zinc; ZnONPs, zinc oxide nanoparticles

FTIR test was performed to characterize the specific chemical bonds in the prepared nanofibrous mats, as shown in Figure 2a. A peak in the wavelength of 3100–3300 indicates the amide and hydroxylation bond of the chitosan functional groups. Also, with the addition of nanoparticles to the chitosan nanofibrous, the presence of peaks 1573–1633 and 3419 indicate the binding of silver and zinc ions to the chitosan functional groups, respectively. Elemental analysis by X‐ray energy spectroscopy showed silver ions in the scaffolds, which confirmed silver in the samples with map and spot analysis images of these scaffolds (Figure 2b,c).

**FIGURE 3:**
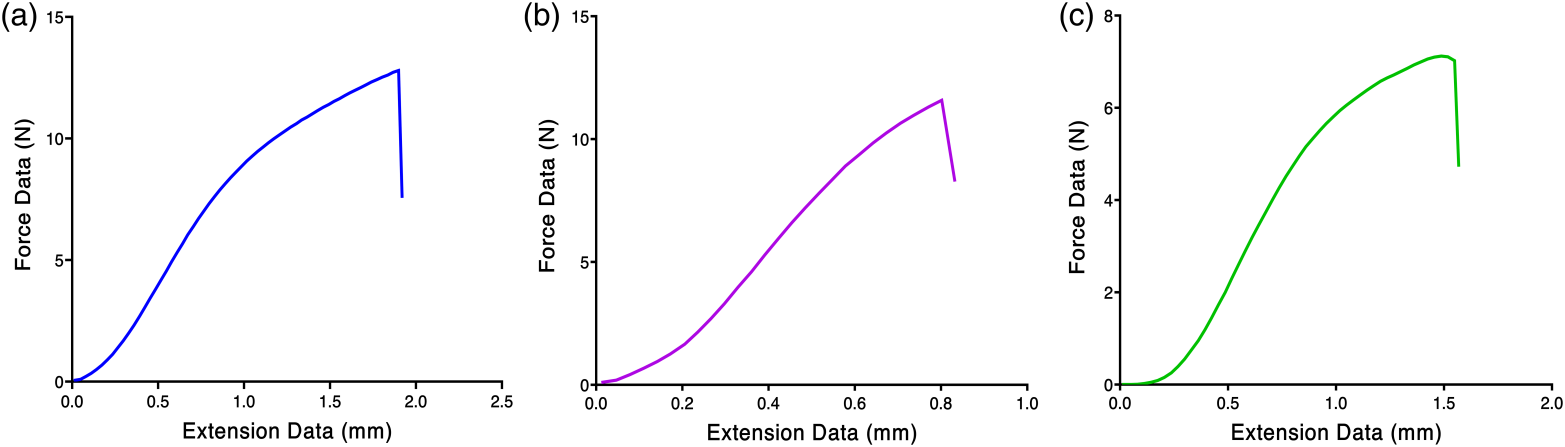
Tensile diagram of a composite scaffold containing: (a) AgNPs, (b) ZnONPs, (c) AgNPs‐ZnONPs

It was found that the ultimate tensile strength at fracture point for nanofibrous mats adorned with Ag, ZnO, and Ag/ZnO NPs are 8.78 ± 8.2, 6.08 ± 5.4, and 5.75 ± 1.8 N, respectively (Figure 3). TPP was used to cross‐link the scaffolds which have the most negligible toxicity to cells. Dissolved tri‐phosphate in deionized water formed OH and P_3_O_10_ ions present in the TPP solution.^33^, ^34^ The FTIR test results of the scaffolds showed that peaks of 1251 indicate the formation of an interaction between the TPP phosphate groups and the chitosan amine groups. As is well known, chitosan is a fragile natural polymer, whereas PEO is a flexible polymer.^18^ Accordingly, as expected, the blend of chitosan and PEO showed sufficient flexibility. Based on the tensile test results, the incorporation of nanoparticles into this blend of nanofibers and nanocomposite fabrication leads to a significant increase in nanofibrous mats' tensile properties and strength.

### 2.3 Antibacterial assessment

**FIGURE 4:**
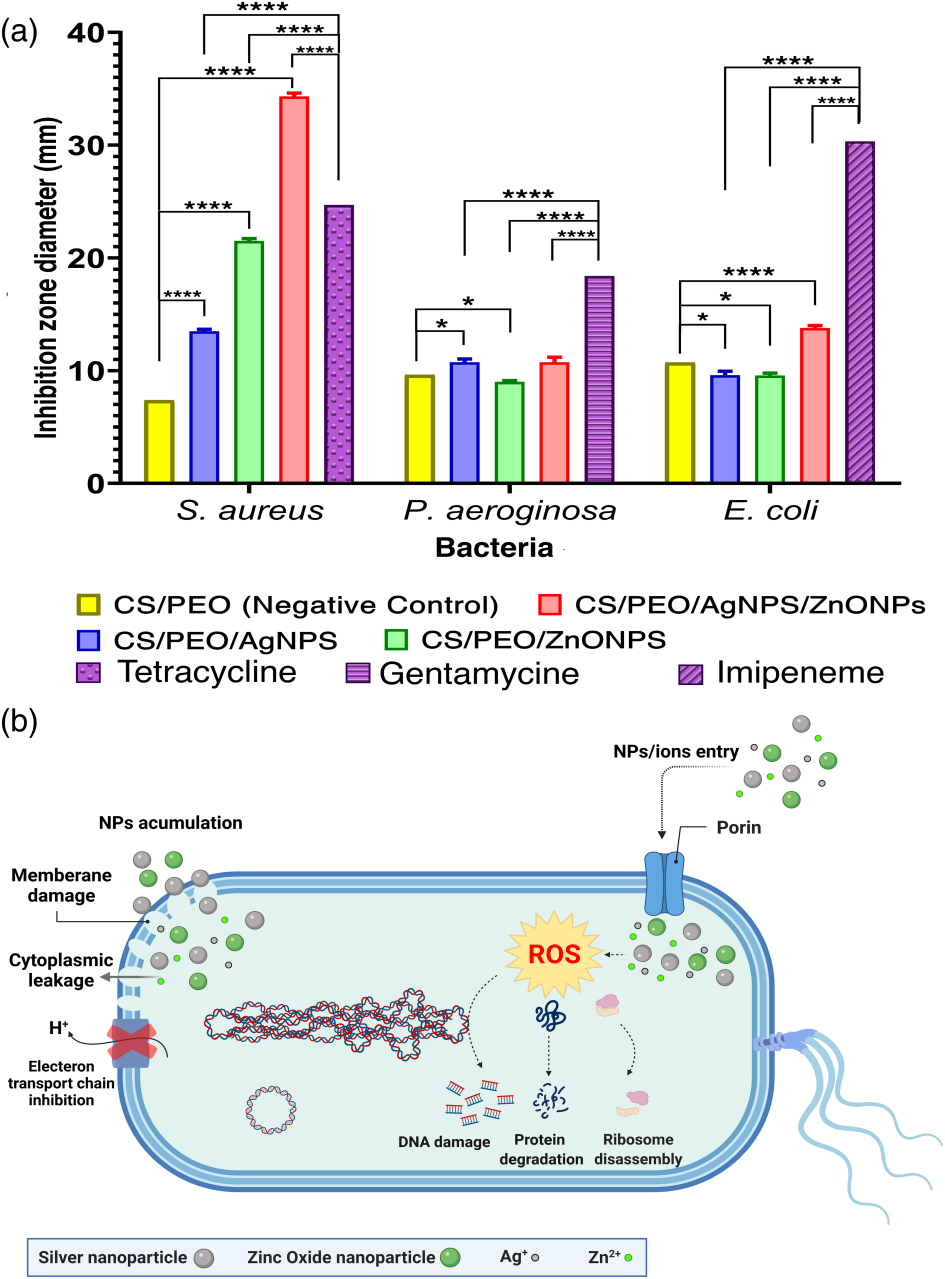
(a) The antibacterial effect of the prepared nanofibrous mats against *Staphylococcus aureus*, *Pseudomonas aeruginosa*, and *Escherichia coli*. Inhibition zone of the various contents of CS, AgNPs, ZnONPs, and AgNPs‐ZnONPs mats compared with selected antibiotic(Ampicillin, Gentamicin, and Penicillin), (b) schematic antibacterial mechanisms of silver and zinc oxide nanoparticles (****p < 0.0001; *p < 0.05)

An antibacterial assessment was performed using a disk diffusion method for each bacterium. The diameter of the inhibition zone was measured after 24 h of incubation by a caliper. As shown in Figure 4a, prepared nanofibrous mats have antibacterial properties compared to the selected antibiotic. In the disk diffusion test, the inhibition zone diameter was employed as an indicator of antibacterial activity of scaffolds; According to the inhibition zone diameter of mats containing silver, zinc oxide, and mixtures of them against Gram‐positive *S. aureus* was more significant than the other two Gram‐negative bacteria. As expected, the Gram‐positive bacterium is more sensitive to these mats. By adding silver nanoparticles, the inhibition zone was formed for all three bacteria. The sensitivity of *S. aureus* was more than the other two bacteria by measuring and comparing the inhibition zone diameter (Figure 4a). Kasithevar et al., by examining the antibacterial effect of silver nanoparticles for some clinical strains isolated from surgical wound infections, including *S. aureus* and *P. aeruginosa*, reported the strong antibacterial effect of this nanoparticle, especially on *S. aureus*.^35^ Other studies have demonstrated the antibacterial activity of silver and zinc oxide nanoparticles with Gram‐positive and Gram‐negative bacteria.^36^, ^37^, ^38^, ^39^, ^40^ Antibacterial activity of metallic nanostructures has been proven to date. Since nanoparticles have shown the lowest toxicity levels in the life cycle and ecosystem, they can be an appropriate choice for the fight against microorganisms.^2^, ^25^ To investigate the synergistic effect of silver and zinc oxide nanoparticles, concentrations of silver and zinc oxide nanoparticles that were more biocompatible were loaded into the composite scaffold. According to the results of the antibiogram test in the present study, it was found that the presence of both nanoparticles in the composite scaffold has a more effective antibacterial effect against all three bacteria compared to the antibiotic and CS/PEO control disks (Figure 4). The schematic antibacterial mechanisms of silver nanoparticles and zinc oxide nanoparticles have shown in Figure 4b.^25^

### 2.4 Antioxidant activity

**FIGURE 5:**
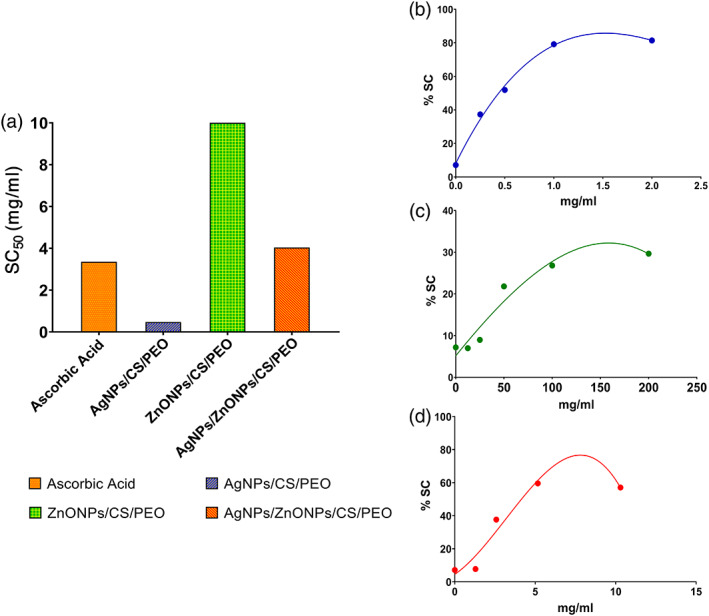
DPPH methods evaluated the antioxidant properties of prepared scaffolds. (a) relative SC_50_ of prepared nanofibrous mats. (b) AgNPs/CS/PEO, (c) ZnONPs/CS/PEO, (d) AgNPs/ZnONPs/CS/PEO

2,2‐Diphenyl‐1‐picrylhydrazyl (DPPH) methods evaluated the antioxidant properties of prepared nanofibrous mats are shown in Figure 5. A DPPH assay was employed to measure the free radical scavenging capacities. The electron or hydrogen donor scaffolds that quench and stabilize DPPH to DPPH‐H are the best at scavenging. Indicates that scaffold contains silver nanoparticles and composite scaffold showed a dose‐dependent scavenging potency similar to butylated hydroxytoluene (BHT) and ascorbic acid (*p* < 0.005), and scaffold contain zinc oxide nanoparticles demonstrated the lowest scavenging activities (*p* < 0.001). When the skin is damaged, large amounts of ROS are produced in the inflammatory phase of wound healing, resulting in biological damage, including degradation of lipids, proteins, nucleic acids, and ultimately cell death, which disrupts the wound healing process. The use of antioxidants can effectively help with enzymatic repair and improve metabolism.^41^ Many studies have shown that nanomaterials are a reliable source of antioxidant activity.^3^, ^42^

The DPPH free radical‐scavenging activity of the scaffolds at different concentrations was evaluated and compared to ascorbic acid and BHT used as a standard. Results indicated that AgNPs/CS/PEO exhibited antioxidant ability against DPPH in a dose‐dependent manner (Figure 5). The highest activity was recorded with AgNPs/CS/PEO and Ag‐ZnONPs/CS/PEO, with the lowest SC_50_ value in scavenging the DPPH (0.47 mg/ml).

### 2.5 Cell viability and hemocompatibility

**FIGURE 6:**
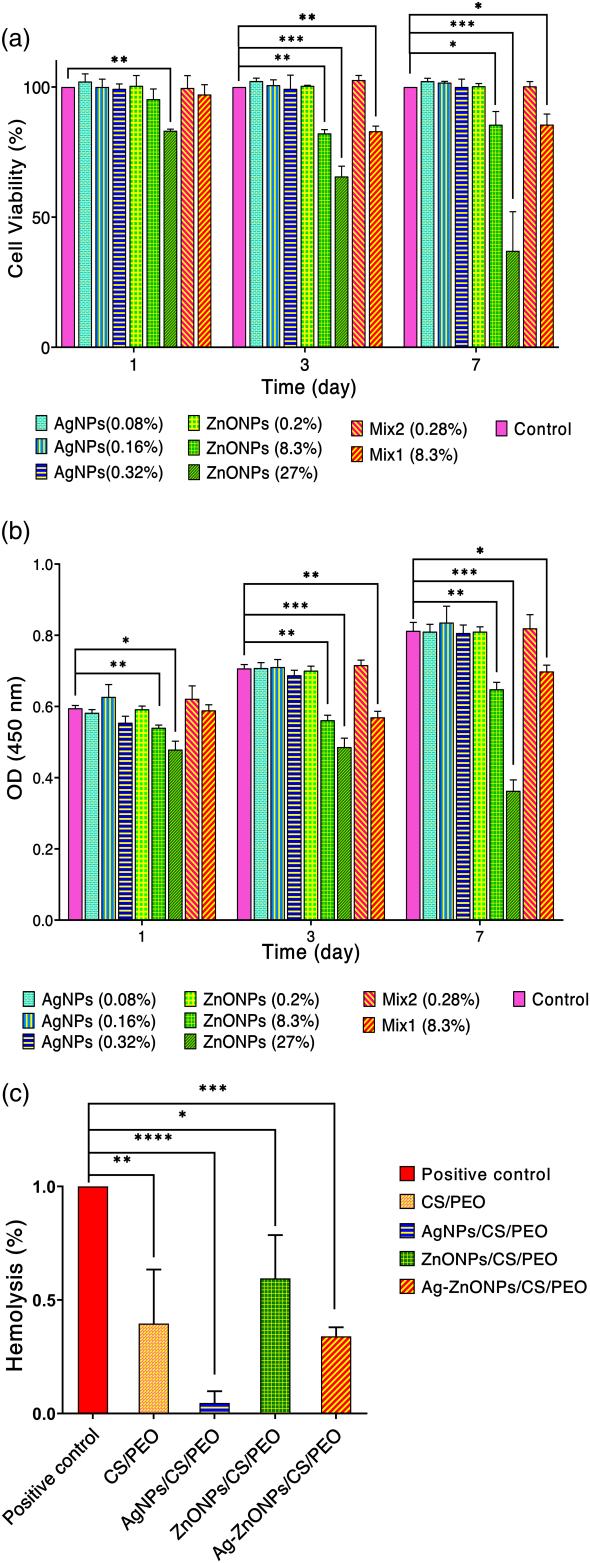
(a) Biocompatibility and viability of fibroblast cells using MTT. (b) Fibroblast cells proliferation and viability assay using CCK‐8 (*p = 0.02, **p = 0.002, ***p < 0.001). AgNPs represent chitosan/polyethylene oxide scaffolds containing silver nanoparticles containing of 0.08, 0.16, and 0.32% w/v. ZnONPs represent chitosan/polyethylene oxide scaffolds containing zinc oxide nanoparticles containing 0.2, 8.3, and of 27% w/v. Samples Mix1 and Mix2 represent chitosan/polyethylene oxide scaffolds containing both silver and zinc oxide nanoparticles containing 0.28% w/v (0.20% ZnO + 0.08% Ag) and 8.38% (0.83% ZnO + 0.08% Ag). (c) The hemolysis ratio of different sample

To assess the cell viability of the fibroblast cells against the nanofibrous mats, two different analysis including MTT and CCK‐8 assay were employed.^43^ According to GB/T 16886.5–2003 (ISO 10993‐5: 1999), samples with cell viability higher than 75% can be counted as noncytotoxic.^44^ According to the OD of samples and standard curve (Figure S1), cell proliferation and cell viability have similar pattern, confirming that the MTT assay results (Figure 6a,b). As can be seen, for all time points the viability of Ag/Cs/PEO are similar to the control group, indicating that silver NPs did not affect the viability in the employed concentration. Regarding the presence of zinc oxide NPs, the result demonstrated that cell viability of the ZnONPs/Cs/PEO samples drops significantly when concentration of ZnONPs enhanced. In this group, nanofibrous mats containing high concentration of ZnONPs (27% w/v.) showed decreased cell viability in the all‐time point. For the concentration of 8.3 wt%, the cytotoxic effect was achieved after 72 h. In contrast, nanofibrous mats containing 0.2% w/v. of ZnONPs, exhibited the cytocompatibility and cell viability similar to the control group in the all‐time point.

For the dual nanoparticles (mix of Ag and ZnONPs), the cell viability of mix 1 was similar to the control group for all time point. Higher concentration of mix NPs (8.38% w/v) the viability reduced upon time. Besides, the cell viability of mix 1 was higher than mix2, indicating concentration dependence of cytotoxicity. The results of the MTT assay and CCK‐8 showed that the optimum ZnONPs concentration in the nanofibrous mats is 0.2% w/v.

According to the American Society for Testing and Materials (ASTM F 756‐00, 2000), each material can be classified into three different categories: hemolytic (hemolysis over 5%), slightly hemolytic (between 2% and 5%), and non‐hemolytic (below 2%). In our study, the hemolytic property of scaffolds was further determined for each nanofibrous mat. The maximal hemolysis percent were detected for CS/PEO mat (3.768%) after contacting erythrocytes over 60 min with prepared nanofibrous mats (Figure 6c). Hemolysis study is an essential test for biomaterials. The blood compatibility results showed that the scaffolds containing AgNPs and ZnONPs had a lower hemolysis effect on human RBC than the scaffold, which had no nanoparticles. These results are in agreement with previous litratures.^45^, ^46^, ^47^, ^48^

### 2.6 Wound‐healing assay

**FIGURE 7:**
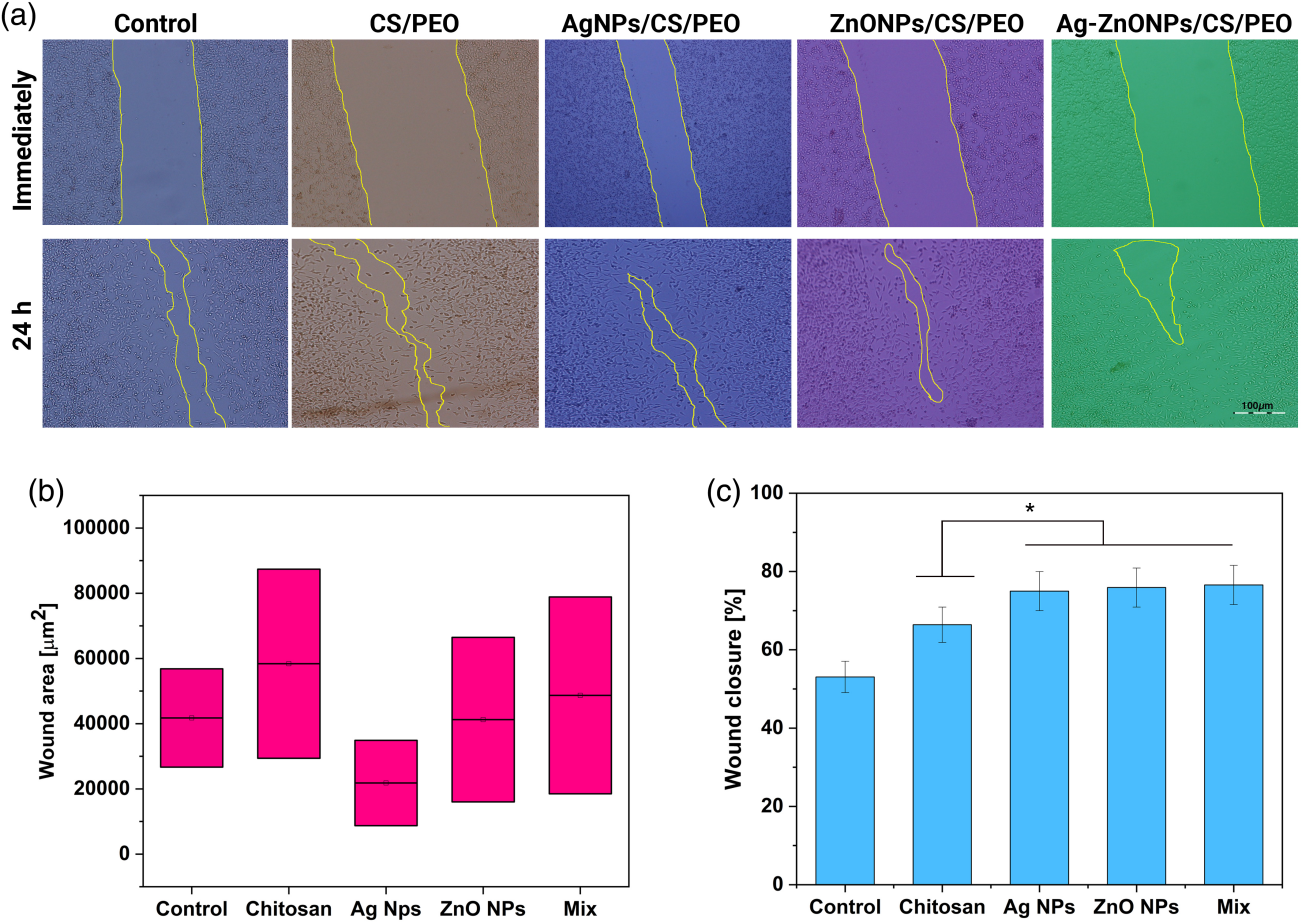
(a) Representative bright‐field images show HDF cell migration after the scratch at time 0 and after 24 h of sample incubation compared with controls. (b) The wound area is expressed as the remaining area uncovered by the cells. The scratch area at time point 0 h, and after 24 h for the samples. (c) Wound closure is expressed as the percentage of the closure of the scratched gap after 24 h. Results are the means of three measurements (*p < 0.05)

To assess cell migration, cell polarity, matrix regeneration, the wound‐healing assay was employed.^49^ This method uses various cells; in this study, the cell monolayer scratching method was used to investigate the effect of chitosan nanofibrous containing the nanoparticles on cell migration of fibroblast cells (L929). In many studies, progressed migration and proliferation of skin cells have been reported due to the treatment of silver and zinc oxide nanoparticles.^50^, ^51^ We found that wound healing was observed up to 24 h after pipette tip scratching. Significant fibroblast migration and proliferation were observed on the wound margin, and after 24 h, the wound healed almost completely. As shown in Figure 7a,b, the migration and proliferation of fibroblast cells at the wound area indicate the ability of chitosan nanofibrous containing silver and zinc oxide nanoparticles to accelerate the wound healing process compared to chitosan scaffolds and controls.

According to Figure 7c, the percentage of wound closure increases with time increase after exposure to nanofibrous mats, especially after 24 h compared to control more than one and a half times. These results show that chitosan nanofibrous can increase the wound healing effect. With the addition of nanoparticles to the nanofibrous mats compared to the control group, this increase is considerably more significant.^50^, ^51^

## 3 DISCUSSION

**FIGURE 8:**
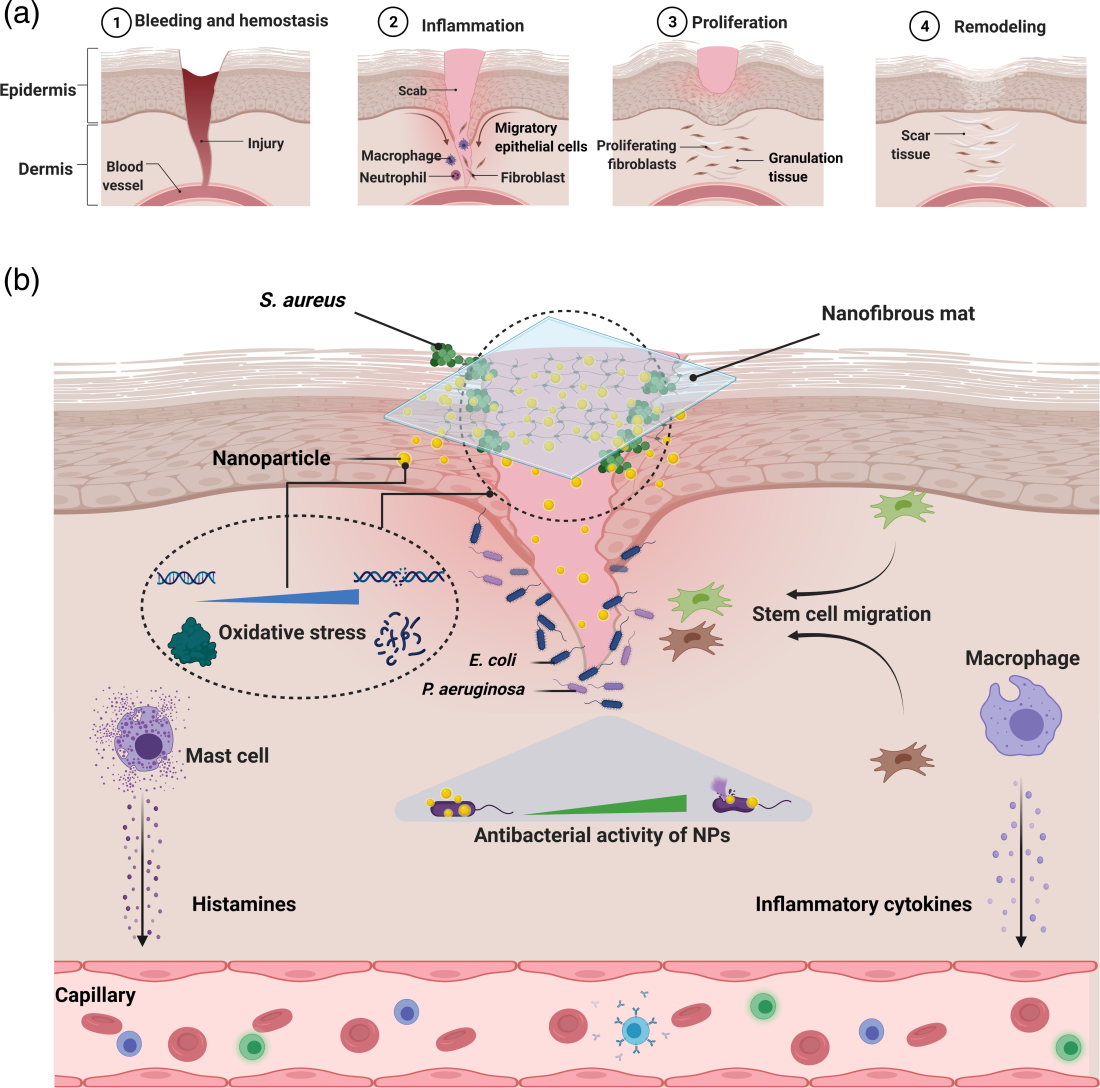
Scheme of (a) stages of wound healing. (b) Schematic illustration for the nanofibrous composite mats with antibacterial properties and trigger the cell migration for wound healing

Wound maintenance is crucial, particularly in diabetic patients and burn injuries. After creation, the wound is part of the skin, and microorganisms proliferate at the wound site. The wound healing process initiates depending on its location, the type of the wound, and wound management. The healing process may be interrupted by both external and internal agents. The internal prerequisite process in wound healing after releasing cytokines in the migration and proliferation of cells. Bacterial infections and reactive oxygen species are important disturbing issues in the wound healing process and can extend each phase.^52^ The process of wound healing comprising four stages of hemostasis, inflammation, proliferation, and remodeling is represented in Figure 8.^53^ Formation of infection in the wound site by external pathogens like *P. aeruginosa*, *S. aureus*, and *E. coli* can undesirably influence the initial phases of the wound healing process via inadequate improvement of tissue granulation. Advanced wound healing antibacterial materials can kill the bacteria multiplied at the wound site and assist in reducing wound‐related complications and accelerated wound healing and may decrease the time of healing.^54^ Our results showed the synergic antibacterial effect of prepared nanofibrous mats against the Gram‐negative and ‐positive bacteria.

Likewise, after creating the wound, oxidative stress damages DNA and proteins in the inflammatory phase because of large amounts of generated ROS. Oral or topical administration of antioxidant materials can avoid oxidative stress by modulating the ROS.^41^, ^55^ It has been shown that nano biomaterials are a reliable source for wound healing remedies and may be effective in wound management. The antioxidant results of the prepared nanofibrous mats have shown their high antioxidant properties. This ability causes the elimination of free radicals at the wound site by the prepared nanocomposite mats. Our results suggested wound healing process can be accelerated through antioxidant, antimicrobial of prepared nanofibrous mats.^56^

The results showed the prepared nanofibrous mats adorned with silver and zinc nanoparticles possess the considerable antioxidant ability and synergic antimicrobial activity. Consequently, these characteristics can synergistically prompt wound healing processes in acute and chronic forms. Figure 8 shows a schematic illustration for the nanofibrous mats with antioxidant activity and synergic antibacterial properties to stimulate wound healing.

As reported, zinc oxide and silver nanoparticles cause bacterial death by acting on the bacterial cell wall and membrane, inhibiting protein and DNA synthesis, and disturbing the antioxidant system.^25^, ^38^, ^57^, ^58^, ^59^ In our study, adding these two nanoparticles to the chitosan scaffold showed a synergistic effect, indicating that scaffolds containing both nanoparticles were more efficient. EDAX map and spot analysis results confirmed the presence of silver and zinc oxide nanoparticles. Zinc nanoparticles have shown their antibacterial effects by reducing bacterial binding and survival on surfaces, disrupting and damaging the bacterial cell wall, increasing permeability, and inducing oxidative stress by penetrating bacteria.^60^, ^61^, ^62^ Tayel et al. observed that the antibacterial potential of zinc nanoparticles was significantly higher than that of zinc powder, which could be due to the higher surface‐to‐volume ratio of smaller particles than larger particles, which increased their efficiency. It was also reported that Gram‐positive bacteria are more sensitive to these nanoparticles than Gram‐negative bacteria. In this study, the inhibition zone diameter nanoparticles for *E. coli*, *P. aeruginosa*, and *S. aureus* were 21, 17, and 31 mm, respectively. The MICs of these nanoparticles were also announced as 16, 26, and 10 μg/ml, respectively.^63^ The results of the present study, which include an inhibition zone diameter for the studied bacteria of 9.57 ± 0.21, 9.01 ± 0.10, and 21.51 ± 0.20 mm, respectively, and MIC values of 8100 μg/ml, 2500 μg/ml, and 61.87 μg/ml, respectively, are entirely consistent with Tayel results. The results of both studies indicate that *S. aureus* is susceptible to other Gram‐negative bacteria; This difference results from the inhibitory concentration of zinc nanoparticles between Gram‐positive and Gram‐negative bacteria, which can be attributed to the presence of an outer membrane in the cell wall of Gram‐negative bacteria and its absence in Gram‐positive bacteria, which affects the permeability of these bacteria to nanoparticles.^64^, ^65^ In line with these results, in the study of Hu et al., The synergistic effect of two nanoparticles of silver and zinc has been reported.^66^ Also, in other studies, the increase in antibacterial effect following the activity of both silver nanoparticles and zinc oxide has been well evident.^67^, ^68^

In antibacterial studies, high sensitivity of *S. aureus* to composite mats containing the nanoparticles was observed compared to *P. aeruginosa* and *E. coli*. Based on the results of the MTT, and CCK‐8 assay, no toxicity was observed for fibroblast cells for composite mats containing AgNPs in all time points, while ZnONPs‐loaded mats showed concentration dependence toxicity. The cell viability as well as cell proliferation of dual‐loaded nanoparticles in composite mats exhibited high biocompatibility. Accordingly, the nanocomposites are promising scaffolds for cell growth and proliferation. This study simultaneously utilized two nanometals in combination of chitosan nanofibrous which showed accelerated the migration and proliferation of fibroblast cells. Scratches after 24 h of treatment showed significant repair. These results indicated that the chitosan/PEO nanofibers scaffolds containing 8.3% AgNPs‐ZnONPs could be a suitable choice for a wound dressing that facilitates the wound healing process; also, composite dressings, in addition to being more effective in treating wounds, can reduce the cost of treatment.

## 4 MATERIALS AND METHODS

Chitosan (deacetylated ≥90%, viscosity 20.0–500.0 mPas, 1 wt% in 1% acetic acid 25°C) was purchased from Solarbio Life Science (Beijing, China). PEO (900,000 g/mol), deionized water, 3‐(4,5‐dimethylthiazol‐2‐yl)‐2,5‐diphenyltetrazolium bromide (MTT), DPPH, CCK‐8 kit (Sigma‐Aldrich, St. Louis, MO, USA), Dulbecco's modified Eagle's medium (DMEM) (Gibco, Germany), Fetal bovine serum (FBS) (Gibco, Germany), Phosphate buffered saline (PBS), trypsin, penicillin & streptomycin (Sigma‐Aldrich), silver nanoparticle and zinc oxide nanoparticle (Sigma‐Aldrich); Acetic acid (Merck, Germany), Mueller Hinton agar (Merck, Germany) and Tryptic Soy Broth (Gibco, Germany); *E. coli* (ATCC25922), *S. aureus* (ATCC25923), and *P. aeruginosa* (ATCC 27853), fibroblast cell (L929) was purchased from Pasteur Institute of Iran.

### 4.1 Suspension preparation of nanoparticles

Based on similar studies, 100 μg/ml of silver nanoparticle was selected to prepare the suspension of Ag NP.^69^ The stock solution was prepared as follows: 200 μg of the nanoparticle powder was dispersed in 1 ml of Müller Hinton broth and prepared as the primary stock for MIC testing for the bacteria studied.

The solution comprising zinc oxide nanoparticle prepared as following; a specific concentration was considered by default for each studied bacteria based on previous studies. For *S. aureus*, the concentration of initial stock was 1.98 mg/ml: for *E. coli*, 64.8 mg/ml and for *P. aeruginosa*, 20 mg/ml.^63^, ^70^

### 4.2 Broth‐microdilution method

There was no particular antibacterial guideline for nanoparticles. According to standard procedures recommended by the CLSI (Clinical Laboratory Standards Institute), the antibacterial effects of silver nanoparticles were measured by broth microdilution method. Broth microdilution approach with some modifications was employed to determine the minimum inhibitory concentration (MIC) of silver and zinc oxide nanoparticles of the tested microorganisms.

This method was used to determine the MICs different concentrations, including 3.2–100 μg/ml AgNPs and 30.93–990 μg/ml, 1.01–32.4 μg/ml, 0.312–10 μg/ml ZnONPs. For culture on a microplate, 100 μL of Mueller Hinton broth was first added to all wells, 100 μL of the initial concentration of nanoparticle solution was poured into the first well and mixed with culture medium dilution was performed from well A to well F. Then, 100 μL of bacterial suspension with the inoculum concentration equivalent of 0.5 McFarland was added to each well and mixed well with the aid of a sampler. One well was considered as a positive control containing Mueller Hinton broth and bacterial suspension. One well, which contained only the Mueller Hinton broth, was considered as a negative control (NC). This procedure was performed for all three bacterial strains and specific concentrations of nanoparticles with three replications. After a slight rotation of the microplate to smooth the suspension into the wells, it was incubated for 24 h in an incubator at 37°C. After 24 h at 37°C, the turbidity of each sample was determined.

### 4.3 Preparation of chitosan/PEO solution containing silver nanoparticle for electrospinning

To prepare chitosan/PEO solution, chitosan and PEO solutions separately in 80% V/V and 0.5molar acetic acid, prepared respectively. Then, the prepared solutions are mixed to a certain proportion.

To prepare a 3 wt% chitosan solution, first, 0.3 g of chitosan powder was poured into a beaker, then 80% acetic acid was added. The solution was stirred for 16 h at room temperature on a magnetic stirrer at 700 rpm. A 3 wt% PEO solution was prepared similar to chitosan. After adding 0.5 molar acetic acid to 0.3 g of a PEO powder to give a uniform solution, it was stirred for 8 h at ambient temperature at 400 rpm; after separate preparation of these solutions, the chitosan solution and the PEO were mixed at a weight ratio of 9:1 and stirred at room temperature at 400 rpm for 12 h. Then, proper concentrations of silver nanoparticles according to MIC were added to the chitosan solution for each bacterium. Thus, a solution containing 0.16% silver nanoparticle was prepared for *S. aureus*, a solution containing 0.08% silver nanoparticle for *E. coli*, and a solution containing 0.16% silver nanoparticle for *P. aeruginosa*. The prepared solution was then filled into the syringe and put into an electrospinning device with the following parameters: voltage 20 KV, a distance of needle from collector 14 cm, rotation of collector 700 rpm. To achieve nanofibrous mats adorned with silver nanoparticles with appropriate thickness, electrospinning was performed using the obtained parameters for 6 h.

### 4.4 Preparation of chitosan/PEO solution containing zinc oxide nanoparticle for electrospinning

For fabrication of the nanofibers containing zinc nanoparticles, concentrations obtained from MIC zinc nanoparticles for each bacterium were added to the chitosan solution. Thus, a scaffold containing 0.2% zinc nanoparticles was produced for *S. aureus*. A scaffold containing 27% zinc nanoparticles was fabricated for *E. coli*. A scaffold containing 8.3% zinc nanoparticles was fabricated for *P. aeruginosa*. The same settings obtained from the electrospinning of silver scaffold were used for electrospinning, the polymeric solution containing zinc oxide nanoparticles.

### 4.5 Preparation of chitosan/PEO solution containing silver and zinc oxide nanoparticle

To preparing the nanofibrous mats containing the silver and zinc nanoparticles, concentrations obtained from MIC nanoparticles for each bacterium were added to the chitosan solution. Thus, Mix1 scaffold contains 0.28% zinc and silver nanoparticles (0.08% silver nanoparticles and 0.2% zinc nanoparticles), and Mix2 scaffold containing 8.4% zinc and silver nanoparticles (0.08% silver nanoparticles and 8.3% zinc nanoparticles) were obtained.

A 1% TPP solution was used as a cross‐linker to improve the strength of the nanofibrous mats. Specimens were prepared from scaffolds measuring 3 × 2 cm and immersed in TPP solution for 5–15 min, washed with PBS after each step, and dried at room temperature.

### 4.6 Characterization

The morphology and diameter of the nanofibers were examined by scanning electron microscopy (FESEM; Philips XL30) after coating with gold by gold sputtering apparatus. ImageJ software was used to measure the average diameter of nanofibers. X‐ray energy dispersive spectroscopy coupled to FESEM apparatus was used for elemental analysis of prepared nanofibrous mats to confirm nanoparticles' presence in the nanofibrous mats. The FTIR was performed in the 4000 to 400 cm^−1^ region using Bruker Tensor 27 IR to investigate the characteristic bonds in the nanofibrous mats. A tensile strength test was used with SANTAM universal testing machine (STM‐1 model) to evaluate the tensile strength of scaffolds. For this purpose, 30 × 10 mm specimens were cut from scaffolds with a diameter of 200 ± 20 μm and used for this test. A tensile strength test was performed using three samples of each scaffold, and the results were presented as mean.

### 4.7 In vitro antioxidant activities

The antioxidant activities of the prepared scaffolds were evaluated using DPPH radical‐scavenging activity. The DPPH radical‐scavenging activities of CS/PEO, AgNPs/CS/PEO, ZnONPs/CS/PEO, and AgNPs/ZnONPs/CS/PEO were determined as described as reported in^71^ with slight modification. Briefly, a volume of 1 ml of methanolic dilutions equivalent volumes of prepared scaffolds was mixed with 1 ml of 80 μg/ml of DPPH as a free radical source. The mixtures were then kept for 30 min in the dark at room temperature (25°C ± 1). The lower absorbance of the reaction mixture indicated a higher DPPH radical‐scavenging activity. Neutralization of DPPH was measured against the NC at 517 nm by a Shimadzu UV‐1800 spectrophotometer according to the following equation:

$$\%\mathrm{SC}=\left(\frac{A_{\mathrm{NC}}-A_{\text{tsample}}}{A_{\mathrm{NC}}}\right)\times 100$$

NCs consisted of all the reagents except the antioxidant components. BHT (0.5–128 μg/ml) and l‐ascorbic acid (0.5–128 μg/ml) were used as standard antioxidant agents. The SC50 (scavenging capacity‐50) is the nanoparticle concentration causing 50% neutralization effects. The SC50 was determined from the equation of the best‐fitting linear or non‐linear regression curve plotted from the scavenging percent versus oil concentrations. All experiments were recorded three independent times, and the mean values ± SE were reported.^72^

### 4.8 Antibacterial activity of nanocomposites

The antimicrobial activity of nanocomposite scaffolds was evaluated by the disk diffusion method against the two species of Gram‐negative bacteria (*E. coli*, *P. aeruginosa*) and one species of Gram‐positive bacterium (*S. aureus*). The microbial culture medium was prepared according to the manufacturer's protocol and poured into 10 cm plates, followed by separate preparation of a suspension of 0.5 McFarland bacteria. Sterilized cotton swabs from the suspension prepared on plate surface containing Mueller Hinton agar were cultured uniformly. The scaffolds were prepared by incubating the specimens in a 37°C incubator for 24 h, and then the inhibitory areas around the scaffolds were measured.

### 4.9 Cell viability

The MTT assay was done to determine the fibroblast viability on the prepared nanofibrous mats. The cells were cultured in DMEM medium supplemented with 10% fetal bovine serum and 1% penicillin/streptomycin antibiotic under standard conditions of 37°C and 5% CO_2_. Each experiment was performed three times at the specified culture durations of 1, 3, and 7 days in a 96‐well plate (3 × 10^3^ cells/well) as reported previously.^21^, ^22^ Finally, the microplate reader (ChroMate‐4300, FL, USA) at 570 nm was used to measure the absorbance of the samples. For better assessment and evaluating biocompatibility, after assessing the cytotoxicity with MTT assay, we evaluated the viability as well as cell proliferation using cell counting kit‐8 (CCK‐8) using fibroblast (L929) cells, respectively. CCK‐8 assay is a more sensitive assay than any other tetrazolium salts (e.g., MTT, MTS, or XTT). Such analysis allows more sensitive colorimetric assays for the indication of the number of viable cells in the proliferation assays and cytotoxicity.^43^

Cells were seeded on nanofibrous mats and well as controll in 96‐well plates at 5 × 10^3^ cells/well. Cell proliferation was investigated after 1, 3, and 7 days using CCK‐8 assay. To create a calibration curve, after preparing the wells with known numbers of viable cells, 10 μL of the CCK‐8 solution was added to each well of the plate. The plates were incubated for 1 to 4 h in the incubator, and the absorbance were measured at 450 nm using a microplate reader. The calibration curve prepared using the data obtained from the wells that contain known numbers of viable cells (Figure S1).^43^ The one‐way analysis of variance (ANOVA) with a statistical significance level of less than 5% (*p*‐value < 0.05) was used to compare the cytotoxicity data.

### 4.10 Hemolysis

CS/PEO, AgNPs/CS/PEO, ZnONPs/CS/PEO, and AgNPs/ZnONPs/CS/PEO nanofibrous mats were tested for their efficiency on human erythrocytes. The blood was obtained from the adult volunteers under consent and placed in heparinized tubes. The PBS (phosphate buffer saline) and Triton Xtm‐100 were used as negative (0% hemolysis) and positive (100% hemolysis) control, respectively. Centrifugation was done at 1500 rpm to segregate the RBCs (red blood cells) pellet from the blood (for 15 min at 4°C) followed by three times supernatant evacuation by PBS, and RBC was diluted with PBS. The specimens (1 cm × 1 cm) were incubated in 800 μL PBS at 37°C for 60 min. Then 200 μL diluted RBC was added to each sample and incubated at 37°C for 30 min. Then, the resulted solutions were centrifuged at 1500 rpm for 15 min, and the absorbance (545 nm) measurement of the supernatant was determined by the spectrophotometer (Shimadzu UV‐1800). Hemolysis cells percentage was calculated as follow:

$$\text{Hemolysis}\;\left(\%\right)=\frac{{\mathrm{OD}}_{s}-{\mathrm{OD}}_{n}}{{\mathrm{OD}}_{p}-{\mathrm{OD}}_{n}}\times 100$$

OD_s_, OD_n_, and OD_p_ were the absorbances of sample, negative, and positive controls. All the hemolysis experiments were performed in triplicate.

### 4.11 In vitro wound‐healing assay

The wound healing potential of the realized formulations was assessed by in vitro wound‐healing assay.^73^ To this aim, fibroblast cells (L929) were seeded at a density of 5 × 10^4^ cells/ml on six‐wells to obtain a monolayer of cells. Then, a scratch was made across the middle of each well using a sterile 1000 μL pipet tip, and the plates were washed twice with PBS to remove the detached cells. A new medium was added, and approximately 1 × 1 cm sized of prepared scaffolds were placed over the scratched area. A control without any samples was also included. Scratches were observed and imaged under the microscope (Nikon Inverted Fluorescent Microscope) immediately after the wounding procedure and after 24 h of incubation. The wound area was calculated using the Image J public domain software. The percentage of wound area reduction or wound closure, expression of the cell migration rate, can be expressed as:

$$\text{Wound Closure}\%=\left(\frac{A_{0}-A_{t}}{A_{0}}\right)\times 100$$

A_0_ is the area of the wound measured immediately after scratching, and A_t_ is the area of the wound measured 24 h after the scratch is performed. The closure percentage increases as cells migrate into the scratch over time.

## 5 CONCLUSION

In the presented work, the results showed the chitosan nanofibrous mats adorned with silver and zinc nanoparticles possess considerable antioxidant ability and synergic antimicrobial activity. Moreover, the enhanced activity of the Zn/Ag combination with chitosan is also attractive as it improves the overall performance, bringing out the potential of single‐device antibiotic combinations with synergic antibacterial and antioxidant properties. In antibacterial studies, high sensitivity of *S. aureus* to composite mats containing the nanoparticles was observed compared to *P. aeruginosa* and *E. coli*. Further investigation of cytotoxicity assessment revealed no toxicity for fibroblast cells, while the viability of fibroblast on composite mats was significantly increased, indicating that not only the composite mats had no toxic effect, but also is a promising scaffold for cell growth. This study showed the simultaneous use of these two nanoparticles in the combination of chitosan nanofibers, accelerating the migration and proliferation of fibroblast cells. Scratches after 24 h of treatment showed significant repair. These results indicated that the chitosan/PEO nanofibers scaffolds containing 8.3% AgNPs‐ZnONPs could be a suitable choice for a wound dressing that facilitates the wound healing process; also, composite dressings, in addition to being more effective in treating wounds, can reduce the cost of treatment.

## CONFLICT OF INTEREST

The authors declare that they have no conflict of interest.

## AUTHOR CONTRIBUTIONS

**Mitra Bagheri:** Investigation (equal); methodology (equal); writing – original draft (equal). **Majid Validi:** Methodology (equal); validation (equal); writing – review and editing (equal). **Abolfazl Gholipour:** Funding acquisition (equal); project administration (equal); supervision (equal). **Pooyan Makvandi:** Formal analysis (equal); investigation (equal); methodology (equal); writing – original draft (equal); writing – review and editing (equal). **Esmaeel Sharifi:** Investigation (equal); methodology (equal); project administration (equal); supervision (equal); writing – review and editing (equal).

## Supporting information


**Appendix** **S1**. Supporting information.
