# Supplementary material for: Chitosan nanofiber biocomposites for potential wound healing applications: Antioxidant activity with synergic antibacterial effect
Source: Bioeng Transl Med. 2021 Sep 16;7(1):e10254. doi: 10.1002/btm2.10254 (PMC8780905; doi:10.1002/btm2.10254)
Supplement: Supplementary file 1 — Appendix S1. Supporting information. [file BTM2-7-e10254-s001.docx]

Table S1. Wave numbers are observed and their associated bonds in scaffolding.

| **Substance** | **Bond type** | **Wave number (cm^-1^)** |
| --- | --- | --- |
| Chitosan | N-H , O-H | 3100-3300 |
| Amide I | C=O | 1680 |
| Amide II | N-H | 1586 |
| Silver | Ag-N  Ag-O | 1573-1633 |
| zinc | Zn-N  Zn-O | 3419 |
| phosphate | P=O | 1000-1250 |
| Ammonium | NH_4_^+^ | 1411-1576 |

Table S2. DPPH radical scavenging activity for prepared nanofibrous mats and positive controls.

| Sample | Vit C | BHT | AgNPs /CS/PEO | ZnONPs/CS/PEO | AgNPs/ZnONPs/CS/PEO |
| --- | --- | --- | --- | --- | --- |
| DPPH radical scavenging activity [SC50, mg/ml] | 0.03±0.000^a^ | 0.02±0.000^a^ | 0.47±0.004^a^ | >200^b^ | 3.48±0.014^a^ |
| ^a^The SC50 representing scavenging concentration-50 are the nanoparticles concentrations scavenging 50% of DPPH.  ^b^The extrapolated SC50 values for ZnONPs/CS/PEO is 256.197 mg/ml. | | | | | |


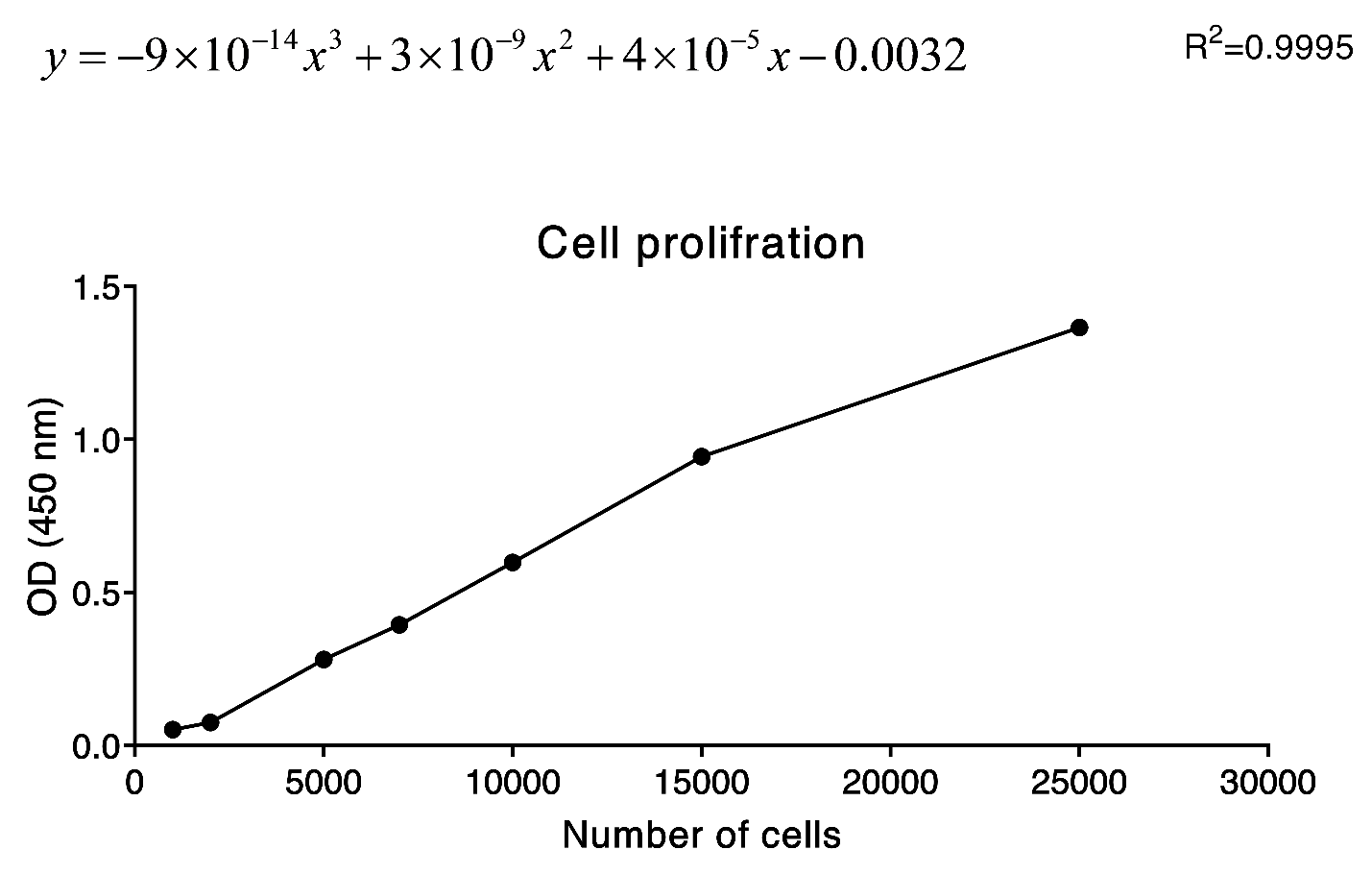


Figure S1. The calibration curve prepared using the data obtained from the wells that contain known numbers of viable cells.
